# Circadian rhythms in cardiovascular (dys)function: approaches for future therapeutics

**DOI:** 10.1038/s44325-024-00024-8

**Published:** 2024-09-23

**Authors:** Margaux Lecacheur, Daniëlle J. M. Ammerlaan, Pieterjan Dierickx

**Affiliations:** 1 Max Planck Institute for Heart and Lung Research, Bad Nauheim, Germany; 2 Cardiopulmonary Institute (CPI), Bad Nauheim, Germany; 3 German Centre for Cardiovascular Research (DZHK), Partner Site Rhine-Main, Bad Nauheim, Germany

**Keywords:** Cardiovascular diseases, Stem cells, Cardiovascular biology, Metabolism

## Abstract

The circadian clock is an evolutionarily conserved time-keeper that regulates physiological processes across 24 h. In the cardiovascular system, several parameters, such as blood pressure, heart rate, and metabolism, exhibit time-of-day variations. These features are in part driven by the circadian clock. Chronic perturbation of diurnal rhythmicity due to shift work or irregular social schedules has been associated with an increased risk of hypertension, arrhythmias, and myocardial infarction. This review discusses the impact of circadian rhythms on human cardiovascular health and the effect of clock disruption on the occurrence of adverse cardiac events. Additionally, we discuss how the main risk factors of cardiovascular diseases, such as obesity, sleep disorders, and aging, affect circadian rhythms. Finally, we elaborate on chronotherapy as well as on targeting the clock and highlight novel approaches to translate our scientific understanding of the circadian clock into clinical practice.

## Introduction

Nearly all forms of life on our planet organize physiological and behavioral processes in an oscillatory fashion within a 24-h cycle. This motion is entrained by the rotation of the earth and the resulting light-dark cycles^1,2^. Together with other external cues, such as food intake or exercise, these so-called *Zeitgebers* (time-givers) allow for anticipation of daily changes in the environment. This is achieved by translating these cues into oscillations and synchronization of internal processes. For instance, higher body temperature and glucose metabolism are associated with greater energetic needs during the wake time of an organism^3,4^. A gene-regulatory network of transcription factors, called core clock factors, underlies circadian rhythmicity^5^. Clock factors regulate clock-controlled genes (CCGs) that, as a result, are expressed in an oscillatory manner. The central or master clock is located in the suprachiasmatic nucleus of the hypothalamus and acts as a central pacemaker that is entrained mainly by light and controls sleep-wake cycles through the secretion of hormones, such as melatonin^6^. Via neural and humoral signals, the central clock relays this information to peripheral organs, thereby synchronizing this information. Peripheral clocks control circadian homeostasis and are present at all organizational levels of the body, including in cells, tissues, and organs^5,7^. Peripheral clocks can also be entrained directly by *Zeitgebers*, such as food and exercise.

In the cardiovascular system, several physiological parameters exhibit time-of-day variations. For instance, blood pressure (BP)^8^, heart rate (HR)^9^, vascular tone^10^, and myocardial metabolism^11^ are regulated in a circadian manner. Importantly, their rhythmicity is essential for proper cardiac function. Indeed, several studies have linked (chronic) perturbation of circadian rhythms to increased occurrence and exacerbation of cardiovascular disorders in both rodents and humans. In humans, these conditions include obesity^12^, hypertension^13^, atherosclerosis^14^, and arrhythmias^15^, and can ultimately lead to myocardial infarction (MI)^16^ and heart failure (HF)^17^. Therefore, many components of modern life, such as shift work^18^, (social) jet lag^19^, irregular sleep patterns^20^, and inconsistent meal times^21^, pose a risk to cardiovascular health in humans.

In this review, we discuss the molecular mechanisms that underlie the integration of external cues into the physiological parameters of (different cell types within) the cardiovascular system. Moreover, we elaborate on how disruption of synchronization between the internal clock and the environment causes disease. To this end, we assess the current state of circadian research by summarizing recent findings from human studies that are supported by murine models. Finally, we highlight novel approaches to study the human cardiovascular clock as well as to design therapeutic strategies (e.g., chronotherapy) for prevention and treatment of cardiovascular diseases (CVDs).

## Circadian rhythms in the human heart

### Molecular framework underlying the (cardiac) core clock

**Fig. 1:**
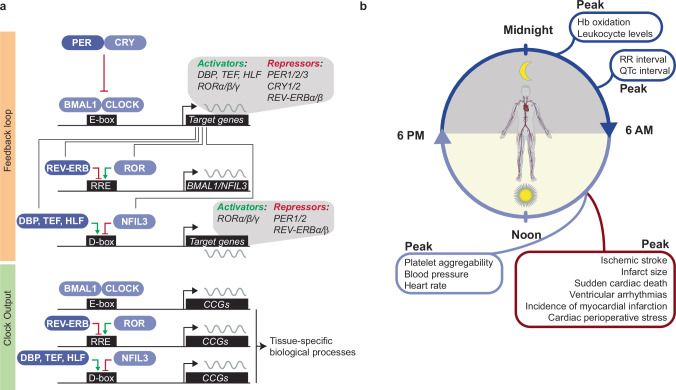
Molecular core clock mechanism give rise to fluctuating (patho)physiological cardiovascular parameters over the course of day. **a** Molecular mechanism of the (cardiac) core clock. *BMAL1* is regulated by REV-ERBs and RORs at REV-ERB/ROR response elements (RREs) on the transcriptional level and by PER and CRY on the translational level. NFIL3, DBP, TEF, HLF exert their effect by binding to D-box motifs within the mammalian genome. The light or dark blue color indicates peak expression of this factor during the day- or nighttime, respectively. **b** Schematic showing the time-of-day variation of crucial physiological cardiovascular parameters (indicated in blue) and the time dependence of adverse cardiac events (indicated in red) in the human body. CCG clock-controlled gene, RRE ROR response element.

Almost all cells within the body possess a molecular circadian clock. This clock system drives transcriptional rhythms of approximately 24 h and comprises multiple factors that act in transcriptional and translational feedback loops (Fig. 1a)^5^. Two of the central players are BMAL1 (brain and muscle ARNT-like 1, encoded by *ARNTL*^22,23^) and CLOCK (circadian locomotor output cycles kaput^24,25^), which, upon heterodimerization, can bind to enhancer box (E-box) elements in promoters, thereby activating the transcription of other core clock genes, including *PER1/2/3* (period1/2/3^26–29^) and *CRY1/2* (cryptochrome1/2/3 ^30,31^). The PER and CRY proteins then form a heterodimer, translocate to the nucleus, and inhibit BMAL1:CLOCK-mediated transcription^32^. Thereby, BMAL1 and CLOCK limit their own expression and create the first negative feedback loop. In a second feedback loop, the BMAL1:CLOCK heterodimer drives rhythmic expression of two nuclear receptor families, REV-ERBs (REV-ERBα/β, also known as *NR1D1/2*) and RORs (RORα/β/γ). These factors, in turn, compete for binding to ROR response elements in the *BMAL1* promotor. While REV-ERBs mediate transcriptional repression, RORs enable transcriptional activation of *BMAL1*^7^. The third regulatory loop includes D-binding protein (DBP), thyrotroph embryonic factor (TEF), hepatic leukemic factor (HLF), and E4 promoter binding factor 4 (E4BP4, encoded by *NFIL3*). These factors either repress (E4BP4) or activate (DBP, TEF, HLF) the transcription of *PER1/2, RORα/β/γ*, and *REV-ERBα/β* by binding to their D-box motifs^33,34^. In addition to autoregulation, the core clock regulates circadian expression of CCGs in a tissue-specific manner (Fig. 1a). This tissue specificity is facilitated by the concerted interplay of core clock factors with tissue-specific transcription factors, such as KLF15, in the heart^35^. In mice, up to 20% of the transcriptome comprises CCGs. In the heart specifically, ~6–10% of genes exhibit rhythmic expression^36–41^. This finding indicates that the circadian clock influences a large percentage of the cardiac transcriptome.

### Systemic diurnal variation in cardiovascular parameters in healthy humans

Cardiac CCGs encode a wide array of proteins that regulate oscillatory physiological processes that facilitate tissue homeostasis. These parameters include HR influenced by, e.g., the cardiac CCG *Hcn4* (Hyperpolarization-activated cyclic nucleotide-gated K^+^ channel 4, responsible for the pacemaker current^42,43^) and electrophysiological parameters (e.g., ion channels through genes such as *Scn5a* (cardiac sodium channel Na_V_1.5)^44^).

In addition, the RR interval, which represents the duration between each cardiac cycle, and the corrected QT (QTc) interval, which represents the length of ventricular depolarization and subsequent repolarization, display a robust diurnal rhythm and peak during the night^45,46^. Other processes associated with time-of-day variations in humans include cardiac metabolism, glucose oxidation [e.g., *Slc2a4*], glycogen synthesis [e.g., *Ppp1r3b*], nicotinamide adenine dinucleotide [NAD^+^] biosynthesis [e.g., *Nampt*^47,48^], the regulation of a number of signaling pathways [e.g., AMPK] [e.g., *Prkaa2*], AKT [e.g., *Akt2*], and mTOR [e.g., *mTor*]; vascular tone; and hematological parameters, such as leukocyte levels and hemoglobin redox status (both peaking at the rest phase) (Fig. 1b)^10,49–51^.

Another example is BP, which steeply increases during the morning, mildly elevates during the early evening, and dips at night. A morning surge in BP and HR is thought to be the result of increased cortisol levels and adrenergic stimulation after waking up^52,53^. Shea et al. showed that intrinsic, clock-related mechanisms also play a role in circadian BP^54^. Individuals who were subjected to a forced 20- or 28-h day regimen still had a 24-h BP rhythm, with the peak in the evening and no major morning peak. This finding suggested that an underlying mechanism, such as the circadian clock, is responsible for 24 h oscillations in BP^54^.

Taken together, these examples highlight that human cardiovascular circadian clocks are tightly linked to essential cardiac physiology. For some processes, specific factors with oscillatory gene expression and protein levels have been identified, while for others, the links between CCGs and rhythmic tissue physiology are yet to be determined. This will aid in both the discovery of tissue functions of CCGs as well as the identification of tissue physiology regulation through CCGs.

## Studying the human cardiac clock

### In vivo

While the molecular mechanisms underlying circadian rhythmicity have been extensively studied in mouse models and human cell lines (discussed later), it is challenging to investigate the clock at this level in humans. Longitudinal sampling is difficult for many organs (e.g., heart), as performing biopsies is highly invasive, and controlling for confounding lifestyle factors (e.g., diet, activity pattern, and biological age) that influence the clock is difficult. Nonetheless, analysis of human samples is necessary to understand how the clock governs circadian cardiac processes in vivo. For instance, a major difference between rodents and primates is the time of activity, with mice being nocturnal while humans are diurnal. A study by the Panda laboratory addressed this issue by comparing rodents and nonhuman primates. The peak of core clock factor expression was shifted by approximately 12 h. This effect was most evident for *BMAL1* and *PER1*, which peaked in baboons^55^ in the evening and morning, respectively, while the opposite was true in mice^41^. The 12-h nocturnal/diurnal switch was less obvious for CCGs, where 47% of the shared oscillators cycled with a < 6 h phase difference between both species. These findings suggest the diverse autonomous temporal organization of clock output regulation. Whether the small phase difference in many CCGs reflect a true species-specific time difference in specific physiological processes is still unclear. Nonetheless, genes encoding important regulators of essential cardiac processes oscillated in both mice and baboons. These included CCGs involved in metabolism (e.g., *Nampt*, *Slc39a14*, *Tecrl*), cardiac remodeling in heart disease (e.g., *Meox1*, *Aqp1, Serpine1*), contractility (e.g., *Tnnt3*, *Mylk3*), and signaling (e.g., *Bmp4*, *Agap2*)^55^. There are some cardiac data available for humans as well. For example, Leibetseder et al. described the rhythmic expression of *PER1/*2 and *BMAL1* in left papillary muscle (muscle connecting ventricle wall with atrioventricular valves) sampled at varying times during orthotopic heart transplantation from cardiomyopathy patients and healthy donors^39^. A time series analysis according to the time of collection showed no difference in core clock gene rhythms between diseased and healthy samples. Here, *BMAL1* and *PER2* expression levels peaked at 10 pm (beginning of rest phase) and 10 am (beginning of lights-on phase), respectively. The peak expression was shifted by ~12 h compared to mice (ZT0 for *Bmal1* and ZT15 for *Per2*^36^), indicating that the human data were in line with the previously mentioned diurnal baboon data. More recently, using RT‒qPCR analysis, McTiernan and colleagues reported oscillatory expression of *REV-ERBα* and *DBP* in hearts from brain-dead donors^56^.

The described studies were able to draw conclusions about rhythmicity, but often, it is difficult to determine the circadian phase of obtained human cardiac tissue. In addition, the time of death might not always reflect the circadian phase due to differences in chronotype (an individual’s inclination to sleep, eat, or exercise at specific times). Therefore, it is unclear which genes display circadian expression in human tissues. To solve this issue, different algorithms, such as CYCLOPS^57^, TimeTeller^58^, Tempo^59^, tauFisher^60^, and CHIRAL^61^, have been developed. CHIRAL, developed by the Naef laboratory, can detect the circadian phase of individual human samples. CHIRAL is based on a large amount of publicly available transcriptomic data (GTEx) and the integration of temporal information from multiple tissues in each individual. This revealed circadian gene expression in 46 human tissues, including the heart, where more than 700 genes (~1.2%) oscillated with a 24 h pattern in the left ventricle^61^. This is less than the 6–10% of oscillators found in the mouse heart^36–38,40,41^, which could be explained by differences in technology and circadian analysis algorithms, controlled experimental settings in mice vs free-living humans, and species differences per se. Among the human oscillators were circadian core clock genes (e.g., *PER1/2/3*, *BMAL1*, and *REV-ERBα*), calcium (e.g., *ITPKA* and *CYSLTR2*), and cGMP-PKG (e.g., *ATP2A2/3*, *ADRA2A/B*, and *EDNRB*) signaling genes. The authors also found that the majority of cardiac oscillators were shared between males and females. Nonetheless, a subset of the differentially expressed genes was sex-specific. Male-specific oscillators displayed enrichment in pathways associated with tricarboxylic acid cycle (e.g., *OGDH*), fatty acid oxidation (FAO) (e.g., *HADHA/B*) and glycolysis (e.g., *PFKM* and *PFKP*), whereas female-specific oscillators encompassed those involved in focal adhesion (e.g., *COL1A2*) and ECM-receptor interaction (e.g., *ITGA10*). In addition, male hearts contained more oscillating mRNAs. The authors were also able to show that aging is associated with circadian remodeling, where 148 transcripts lost and 49 gained rhythmicity, respectively, in aged cardiac ventricles. 48 oscillators were either dampened, increased or phase-shifted (see paragraph “Clock perturbations in humans”). In conclusion, their analysis revealed that the human heart displays sexual dimorphisms in circadian rhythms and exhibits age-dependent alterations in transcriptome rhythmicity. This landmark paper now forms a great resource for interrogating which genes and pathways (putatively) oscillate in humans.

However, whether and how clocks in different cardiac cell types interact to synchronize or otherwise regulate each other are unknown. Single-cell or single-nuclei RNA sequencing (seq) of human heart samples might shed light on this. Biopsies taken at different times during the day should therefore be analyzed. Through the use of computational tools, such as CellPhoneDB^62^, NicheNet^63^, CellChat^64^, ConnectomeDB^65^, and LIANA^66^, which elucidate putative interactions between cell types via transcriptomics, potential communication between different cardiac cell types could be revealed. While this has been done in a noncircadian manner^67^, adding the temporal component could unveil additional layers of (circadian) regulation. In addition, it is not clear whether physical communication between cardiac cell types is necessary to convey time or whether distinct regions in the heart display different circadian phases. Spatial transcriptomics of cardiac tissues sampled at different timepoints could contribute to understanding whether and how clocks in different cardiac regions are (dis)similar. These tools could show how separate cell types in the heart communicate time to each other in order to stay synchronized.

### In vitro

**Fig. 2:**
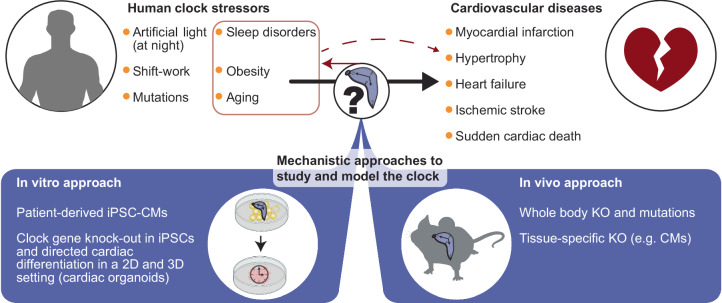
Unraveling mechanisms of cardiovascular disease development upon clock perturbations in humans using in vitro and in vivo models. Several factors, including obesity, aging, shift work, and sleep disorders, can increase the risk for cardiovascular diseases in humans either directly or indirectly via affecting the circadian clock. Conversely, disrupted clocks can lead to sleep disorders, obesity, and premature aging. In vitro, patient-derived iPSCs and clock gene knock-outs in several cardiovascular cell types can be used to investigate human cardiac disease. In vivo, laboratory mice are used to recapitulate these human stressors by the induction of a whole-body or cell type-specific knock-out or by alteration of the light-dark cycles. Additionally, obesity, that is linked to a disturbed circadian clock, can be mimicked by putting mice on a high-fat diet. iPSC: induced pluripotent stem cell; CM: cardiomyocyte.

To study the cell-autonomous nature and molecular mechanisms driving circadian rhythms in human cardiac cells, in vitro models are necessary. One such proxy for the human heart is the use of human embryonic stem cell-derived cardiomyocytes (hESC-CMs), which can be sampled at high temporal resolution to interrogate circadian gene expression patterns or changes thereof in different experimental designs. Indeed, circadian expression of core clock genes and CCGs has been observed in vitro, independent of the systemic context, in hESC-CMs^68^. While human pluripotent stem cells (hPSCs) do not express clock genes in a circadian fashion, oscillatory expression patterns arise during differentiation toward CMs (Fig. 2). Cells that were differentiated for longer durations displayed greater amplitudes, suggesting a link between cell maturation and circadian oscillation^68^. Using hPSC-CMs, human cardiac CCGs, which include the proangiogenic factor *Vascular endothelial growth factor A (VEGF-A)* and *Ubiquitin C* (*UBC*), a factor that mediates the response to stress, were identified. In addition, many other cardiac stress response genes, such as *BNIP3*, *RRAGA*, *DNAJA1*, and *HSPH1*, were found to oscillate in hESC-CMs, suggesting a putative rhythmic response to stress. Indeed, hESC-CMs that were treated at different timepoints with doxorubicin, a commonly used anticancer drug with known cardiotoxic effects, elicited a rhythmic apoptotic response. Similar results were found in neonatal rat cardiomyocytes^69^ and stem cell antigen-1 (*SCA1*)^+^-positive cells^70^ from the human heart. Together, these findings reflect a rhythmic sensitivity to stress in cardiac cells.

Even though 70–80% of the cardiac volume comprises CMs, they account for only 30–40% of the total number of cells in the human heart^71,72^. Additional cell populations include cardiac fibroblasts, immune cells, endothelial cells (ECs), and vascular smooth muscle cells, all of which could have distinct gene expression profiles and unique sets of CCGs. ECs maintain vascular homeostasis and are involved in coagulation, angiogenesis, and the regulation of vascular smooth muscle cell (VSMC) contraction^73–75^. Studies in cultured ECs revealed that coagulation, which is mediated by *plasminogen activator inhibitor-1* (*PAI-1*) and *thrombomodulin* in vivo, is regulated by the BMAL1:CLOCK heterodimer through transcriptional regulation in vitro. In addition, *CCNA1* and *CDK1*, which are responsible for cell cycle regulation, also oscillate in human umbilical vein ECs (HUVECs) cultured in vitro^74,75^. Other factors associated with circadian rhythmicity and involved in EC metabolism include endothelial nitric oxide synthase (eNOS) and NADPH oxidase (NOX)^76,77^. These enzymes produce nitric oxide (NO) and hydrogen peroxide (H_2_O_2_), which serve as signals to induce dilation or constriction of VSMCs. An in vitro model of human carotid VSMC cultures demonstrated 24-h oscillations of *PER1/2/3, CRY1/2*, *REV-ERBα*, and *BMAL1* mRNA in this cell type as well^78,79^. The rhythmic expression of CCGs has also been described in human-derived saphenous vein pericytes, venous blood immune cells, and SCA1^+^ cells isolated from the human heart^50,70,80^. These findings show that many different cardiac cell types exhibit circadian rhythms. Importantly, in addition to these studies in individual cell types, other studies are needed to elucidate how clocks in separate cell types are coordinated to facilitate circadian cardiovascular homeostasis.

Clock development is coupled with differentiation status^7,68,81^, as previously shown in 2D hESC-CMs, which are still relatively immature cardiac cells^68^. It is tempting to speculate that more mature cells might display greater amplitudes of rhythmicity. With the availability of different cardiomyocyte maturation protocols^82,83^, this hypothesis can now be tested. In addition, stem cells could be used to generate cardiac organoids^84–86^, a system that contains more mature CMs and better reflects the cell type complexity of an in vivo human heart. Additionally, efficient endogenous genome editing tools have been developed that allow for tracking of circadian rhythms in cells in real time^87^. This method can be adapted for use with hPSCs to follow the development of human circadian rhythms after differentiation into CMs and subsequent maturation. In conclusion, the advancement and combination of these new methods will allow for the development of good in vitro models for human cardiac circadian rhythmicity.

## Circadian misalignment and cardiovascular pathologies

### Time dependence of adverse cardiac events

To facilitate resilience to abrupt increases in activity after awakening, many cardiovascular parameters exhibit a circadian pattern^6^. Importantly, adverse cardiovascular events also occur more often at certain times of day^88^. These events include MI, sudden cardiac death, and ischemic stroke, which all peak around the sleep-to-wake transition in the early morning (Fig. 1b)^89^. While this time of day coincides with peaks in other parameters, such as BP, shear stress, platelet aggregability, glucocorticoids, and catecholamine levels^90,91^, the importance of cell-intrinsic oscillatory mechanisms in driving this phenomenon has recently become more accepted. Indeed, in mice, tolerance to ischemia/reperfusion was shown to be mediated by the cell autonomous clock, as CM-specific *Clock* mutant mice did not display a 3.5-fold increase in infarct size at the sleep-to-wake versus the wake-to-sleep phase, like control mice do^92^.

The abundance of human immune cells, such as neutrophils, leukocytes, eosinophils, monocytes, and lymphocytes, also displays a time-of-day dependence^50,93–96^. As these cells contribute to inflammation modulation after MI, this rhythmicity could also affect infarct size^94–96^. However, this balance is delicate, with, for example, low neutrophil numbers being important for coordinated resolution of post-MI inflammation and repair but too many neutrophils being detrimental to post-MI outcomes for females^97^, while interestingly the opposite is observed in males^98^. Nonetheless, the different experimental set-up in these studies makes it difficult to assess a true sex-specific difference in immune infiltration after MI. Rhythmicity in circulation and tissue infiltration have also been shown in mice, where increasing numbers of neutrophils and natural killer cells are suggested to boost the humoral immune response^99^. This finding suggested that circadian infarct size is likely determined by a combination of circadian mechanisms in cardiomyocytes themselves, as well as by infiltrating immune cells. Since the immune response upon cardiac damage can be both beneficial and detrimental depending on the timing of the disease^97,98,100^, it will be important to study the mechanisms by which the circadian clock governs these different waves. In addition, disruption of the circadian clock five days after an induced infarction in mice leads to changes in immune cell infiltration (less neutrophils and more macrophages) compared to non-disrupted mice^101^. These results suggest that circadian clock is governing immune infiltration during infarction and that maintaining a proper circadian rhythm could be important for patients that underwent a myocardial infarction (see “Targeting the time” section for details).

In a study from the Staels group, researchers showed that patients who underwent aortic valve replacement in the afternoon had a lower incidence of major adverse cardiac events 500 days after surgery than patients who underwent surgery in the morning^102^. The authors directly linked this to variations in the expression of the circadian nuclear receptors *REV-ERBA* and *B*, and their downstream effector *P21*^102^. This emphasizes the urgency of considering the time of day to minimize the risk of adverse cardiac postsurgery events. In conclusion, these studies show that the circadian clock and (adverse) CV events are tightly linked. It is therefore important to understand how the cardiac clock governs essential cardiac processes and to not disturb it.

### Clock perturbations in humans

Human life is associated with a variety of behavioral, environmental, and genetic perturbations that contribute to circadian misalignment. This includes exposure to artificial light, shift work, sleep disorders, obesity, and aging, as well as genetic mutations and single nucleotide polymorphisms (SNPs)^103^. Many of these disruptors are also known to affect cardiac physiological parameters, leading to an increased risk of CVDs. However, whether and to what extent these CVD outcomes are driven by clock disruption or direct effects on cardiovascular physiology are not yet fully understood. Here, we elaborate on how different clock perturbations are studied and linked to CV (patho)physiology.

Light desynchrony can be caused by artificial light outside regular sunlight hours (e.g., due to the use of mobile phones or shift work)^104^ or social jetlag induced by inconsistent sleep patterns between working and nonworking days^105^. This leads to disrupted pineal melatonin secretion and, consequently, altered circadian rhythms^106,107^. It is important to note that short wavelengths (~460–495 nm) will have a stronger effect on melatonin reduction compared to longer wavelengths (~620–780 nm)^108^. This suggests that exposure to longer wavelenghths in the evening can improve sleep quality and health^109^. In humans, light at night (especially blue light^110^) is correlated with decreased heart rate variability, increased heart rate at night^111^, and increased incidence of cardiometabolic diseases^112–114^. Shift workers often do not experience the typical BP drop that healthy individuals experience^115^. This leads to morning hypertension and increased cardiovascular risk^115–117^. Moreover, shift work influences HR and leads to an increased frequency of ventricular extrasystole^118,119^. The direct connection between shift work and these physiological parameters was shown by the Scheer laboratory. They induced short-term circadian misalignment in healthy subjects by either rapidly shifting light exposure by 12 h or by imposing a forced desynchrony protocol in which 10 individuals were subjected to recurring ‘28-h days’^120,121^. In both studies, the subjects had elevated arterial BP and inflammatory marker levels, which are known CVD risk factors^120,121^. In line with this, long-term night shifts and rotational shift work have been associated with a higher risk of hypertension, left ventricular hypertrophy, and coronary heart disease as well as more severe outcomes after MI^122–127^. In addition, many rodent studies have shown deleterious effects of desynchronized external light versus internal rhythms on the heart. For example, tau mutant hamsters with shorter circadian periods develop dilated cardiomyopathy with early lethality^128^, and infarcted mice exposed to 10:10 light:dark cycles exhibit worsened cardiac remodeling, leading to accelerated HF progression^101^.

Sleep disorders (including insomnia or narcolepsy) can affect cardiovascular functions, leading to CVDs either directly or via circadian misalignment^129–132^. For example, sleep problems influence BP and obesity, which are known risk factors for CVDs^133^. In addition, insomnia, which is frequently caused by stress, has been linked to an increased risk of HF and acute MI^134,135^. Interestingly, while most sleep disorders have other etiologies, mutations in core clock genes can also be drivers of sleep disorders. For example, *PER2* or *CRY2* mutations were found in patients with familial advanced sleep phase syndrome (FASP)^136,137^. These mutations were validated in cell lines and mouse models, where they recapitulated the circadian phase shift observed in patients^136,137^.

High caloric food intake promotes obesity and clock disruption. In mice, a high-fat diet leading to obesity is responsible for circadian perturbations, such as lengthening of the active phase and clock gene expression alterations^138,139^. This diet-induced obesity leads to complete reprogramming of the circadian transcriptome in the heart, where important metabolism-related genes lose rhythmicity (e.g., *Acadm, Acca*, and *Cpt1a*), and others gain rhythmicity (e.g., *Pdk4a*, *Pfkm*, and *Mlycd*). Moreover, obesity is associated with increased expression of markers of adverse cardiac remodeling (hypertrophy and fibrosis)^140^. In humans, obesity (body mass index greater than 30) disrupts circadian patterns of atrial natriuretic peptide (ANP), B-type natriuretic peptide (BNP), and BP^141^, which potentially predisposes these individuals to HF^142^. In addition, (being) overweight and obesity are strongly correlated with other CVDs, including coronary artery disease, MI, atrial fibrillation, and sudden cardiac death^12,143^. However, whether these effects are mediated by disrupted circadian clocks is unclear. Interestingly, a recent study from the Martino lab has shown that *Clock* mutant mice fed a high-fat diet are resilient to cardiac dysfunction, such as cardiac hypertrophy, left ventricular remodeling, and diastolic dysfunction, despite their underlying obesity and metabolic conditions^144^. This study highlights the links between the circadian clock and resilience to CVDs, in a setting of metabolic perturbation. While obesity can affect the clock, the inverse holds true as well; SNPs in the *CLOCK* and *PER2* genes were observed in obese patients compared to their lean counterparts, suggesting a bidirectional link between clock factor deregulation and obesity^145–147^.

Aging can affect the circadian clock in multiple ways. For example, older individuals tend to have a phase shift in their preferred wakefulness time toward a “morning” chronotype compared to young adults^148^. Interestingly, evening chronotype individuals are associated with increased odds of having poor cardiovascular health due to increased cardiometabolic risk factors, such as type II diabetes, poorer physical activity, and detrimental sleep patterns^149–152^. Additionally, aging is linked to dampening and remodeling of CCG expression and circadian processes in humans^61^. Talamanca et al. reported a similar number of oscillators in young (donors <50 years old) and old (donors >60 years old) human hearts. Although most of the rhythmic genes were shared between young and old hearts, a substantial number of oscillators were lost in older individuals. Programs that lost rhythmicity were enriched for adrenergic signaling (e.g., *CREM* and *KCNQ1*), complement and coagulation pathways (e.g., *ITGAX*), and apelin signaling (e.g., *KLF2* and *MYL4*), all of which are known to play a role in or to be affected by CVDs^153–155^. This age-dependent reprogramming has also been observed in mice and is sex-dependent^156–161^. In mice, aging leads to a reduction in the number of cardiac CCGs, as well as to a very distinct circadian transcriptome^156,162^. Circadian patterns of genes essential for cardiovascular physiology (e.g., *Scn5a*, *Grk5*, and *Kcnn2*) were found to be disrupted by aging in the present study. These findings suggest conserved mechanisms by which aging impacts the cardiac circadian clock across species and show that CCGs are not only tissue specific but also age specific within one organ.

CVDs are the leading cause of death worldwide, with 17.9 million deaths annually^163^ and the different perturbations of circadian rhythmicity described above are now recognized as comorbidities for CVD^164–166^. Nonetheless, whether and to what extent these risk factors influence cardiovascular outcomes via their effect on the clock is unknown^167^ and merits additional research. As all the components are interlinked, determining the exact order of events is often difficult.

### Clock perturbation models for studying the link between circadian rhythms and CVDs: from mice to human cells

#### Systemic/organismal level: Mice

The interaction between the circadian clock and CVDs in humans is most often based on observations that show correlations rather than causality. Owing to the availability of genetic tools, ease of breeding, and possibility for longitudinal studies at an organismal level, mouse models are the preferred choice for mechanistically studying the consequences of clock perturbations on CVDs (Fig. 2). Using experimental mice also allows easier identification of factors that recruit clock proteins to chromatin in varying contexts, such as time, tissue, age, and nutritional status. The abovementioned observations (circadian response to stress, remodeled rhythms with age, and correlation between chronodisruption and increased CVD risk) in mice and humans largely overlap, suggesting substantial conservation across mammals.

To recapitulate humans subjected to whole body mutations or living under altered light:dark cycles, constitutive clock gene KO mice have been generated. While the initial reports on whole body *Clock*^168^ and *Rev-erbα/β*^169,170^ KO did not describe any cardiac phenotypes, the KO of *Bmal1* and *Clock*^*Δ19/Δ19*^ mutation induced premature dilated cardiomyopathy^171,172^ and cardiac hypertrophy^160^, respectively. *Per2* KO mice have been reported to display a contrasting cardiac injury response after MI^173,174^, a discrepancy that could be attributed to a difference in the method used to induce the MI.

To mechanistically elucidate the role of the clock in the heart specifically, researchers have knocked out the core clock factors *Bmal1* and *Clock* in CMs (CBK and CCM mice, respectively), which ablates circadian rhythms and leads to severe cardiac phenotypes, such as dilated cardiomyopathy and hypertrophy^175,176^. While BMAL1 and CLOCK are part of the positive arm of the clock pathway, loss of the nuclear receptors REV-ERBα and REV-ERBβ, which regulate the negative arm of the clock in CMs (CM-RevDKO) (Fig. 1a), leads to a reduction in NAMPT/NAD^+^ levels, a similar phenotype of dilated cardiomyopathy and early death as observed in CBK mice^47,177^. However, although metabolism is deregulated in both of these models, the mechanism is different. While FAO is increased and glucose oxidation, glycolysis, and glycogen synthesis are decreased in CBK and CCM mice^175,176^, CM-RevDKO mice exhibit a reduction in both FAO and glucose oxidation but an increase in glucose uptake^47,177^. Decreased FAO and glucose oxidation in CM-RevDKO mice can be linked to downregulated mRNA expression of *Ces1d*, *Dgat2*, *Cpt1a*, *Gpam*, and *Pdk1/2*, while the mechanisms underlying increased FAO in CBK mice are less clear. Interestingly, we and others found that common *Nampt* reduction in CBK and CM-RevDKO hearts was modulated via an increase in the protein levels of the downstream clock factor and transcriptional repressor E4BP4^47,177^. Indeed, cardiomyocyte-specific knockout of *E4bp4* was associated with increased *Nampt* levels^178^. In addition to cardiac metabolism and contractility, cardiac electrophysiological properties, such as heart rate variability ande RR and QT intervals, are also under circadian control (Fig. 1b). For example, CCM mice exhibit a decreased heart rate^176^, lengthening of the QT interval, and altered cardiac expression of a number of ion channel genes involved in cardiac conductance, such as *Hcn4*, *Kchip2*, *Kcne1*, and *Scn5a*^42,44,179^. To our knowledge, there are no other cardiac-specific clock knockout models available thus far. Nonetheless, these findings highlight the complexity and thus importance of studying these features in distinct cardiac clock model systems.

Another variable that clearly influences the consequences of clock perturbation and that has to be studied in more detail in vivo is sex. This is based on different outcomes after knockout of distinct clock factors in males and females. For instance, male germline *Clock* mutant mice developed age-dependent cardiac dysfunction, while females were protected^180^. Ovariectomizing female *Clock* mutants led to cardiac dilation, suggesting that the profound differences were due to ovarian hormones, such as estrogens. Estrogens were also shown to be cardioprotective in other settings, such as pressure overload-induced cardiac hypertrophy^180,181^, indicating that these hormones can have similar effects on different CVDs. In humans, estrogen was also found to be cardioprotective, a feature that decreases with age as estrogen levels are lower in older females^182^. Therefore, it might be interesting to study whether clock disruption via, for example, shift work has a more profound effect on cardiac function in aged males than in aged females. In contrast to *Clock* mutants, we recently showed that female mice with cardiomyocyte-specific *Rev-erbα/β* DKO died earlier than their male counterparts^47^. These differences could be due to the distinct functions of clock proteins or to the contributions of other tissues to cardiac function since the *Clock*-mutant model is germline, while the *Rev-erbα/β* DKO is cardiomyocyte specific.

The findings from different clock disruption models highlight the complexity of the cardiac circadian clock and the need to study different clock factors, as their phenotypes are only partially overlapping. Given that many CVDs are systemic, further genetic circadian clock KO experiments in other tissues that focus on whole-body physiology as well as the heart are necessary.

#### (Multi)cellular level: Human

Genetic mouse experiments allow us to elucidate the molecular mechanisms by which disturbing the circadian clock leads to CVD, but investigating whether these findings translate to humans is challenging. Therefore, in vitro models constitute a great and complementary alternative. In line with CBK mice, which develop lethal dilated cardiomyopathy^175,183^, KO of *BMAL1* in hESC-derived CMs leads to a cellular phenotype that resembles dilated cardiomyopathy with disruption of sarcomere structures, cellular enlargement, impaired contractility, and arrythmias^184^. These findings confirm the phenotype of CBK mice and suggest that BMAL1 in humans is essential for maintaining healthy cardiac functions. Since the CCM and CM-RevDKO mouse models also exhibit cardiac defects, such as hypertrophy and lethal dilated cardiomyopathy, respectively, it might be interesting to test the function of CLOCK and REV-ERBs in human cardiac differentiation and function. To the best of our knowledge, no KOs of other clock factors in human pluripotent stem cells have been reported thus far.

Overall, these studies showed that molecular clock perturbations can lead to cardiovascular defects (Fig. 2). Disease phenotypes can be studied in more detail in mice, but the in vitro setting of stem cell-derived CMs allows for mechanistic follow-up in a human setting. While many (human-specific) molecular mechanisms remain to be further investigated in detail, the large consequences of clock perturbation open up potential avenues for therapeutic approaches to prevent or cure CVDs.

## Therapeutic applications: circadian medicine

**Fig. 3:**
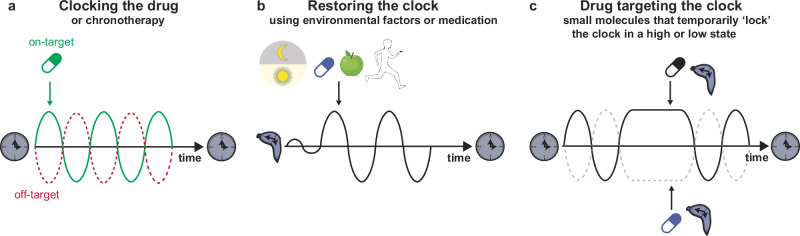
Therapeutic applications for circadian cardiovascular medicine. **a** Timed administration of medication in order to optimize drug efficacy by maximizing on-target effects and minimizing off-target effects. **b** Restoring the perturbed clock using environmental factors, such as strict light-dark schedules, medication, time-restricted feeding or physical exercise. **c** Drugs (small molecules) that target certain clock factors, thereby temporarily ‘locking’ the circadian system in a high or low state.

Circadian rhythms and core clock genes are essential regulators of healthy cardiac functions, and misalignment of circadian rhythms can increase the risk of CVDs (see above). Therefore, it is important to consider the influence of the circadian rhythm on drug absorption, metabolism, and disease pathology. To date, only a few studies have integrated circadian rhythms into treatment schedules for patients. In this section, we will detail the different approaches to understanding the circadian clock to optimize cardiovascular health and treatment, a concept called circadian medicine or chronotherapy (Fig. 3).

### Targeting the time

Taking time into account to improve the efficiency of CVD treatments is an important approach that is beginning to be implemented in the clinic^185–187^. Many different drug prescriptions contain a recommended duration of intake to optimize the beneficial effects of the drug and minimize its adverse effects^188^ (Fig. 3a). Evening dosing (at bedtime) with low-dose aspirin or hypertension medications, such as guanfacine and doxazosin, is more effective at decreasing platelet aggregation^189–191^ or BP to reduce the onset of cardiovascular events in the morning^186,192–194^. On the other hand, morning administration of other drugs, such as verapamil and modafinil, helps to combat daytime sleepiness and potential adverse cardiovascular effects^195–197^. In addition, the use of β-adrenergic or calcium channel antagonists can have protective effects against the morning incidence of cardiac events, such as MI^198^, tachyarrythmias^199^ or myocardial ischemia^200^.

Different gene expression studies^41,55^ have shown that many widely used medications impinge on circadian targets with short half-lives. In circumstances where off-targets of these drugs also display circadian expression profiles, it would be optimal to treat patients when the on-target is highly expressed and off-target expression is low. Therefore, taking time into account for medication could improve treatment efficacy and reduce adverse effects (Fig. 3a).

In addition to the timing of medication, light (a potent *Zeitgeber* of the circadian clock) exposure and affected melatonin can also be considered to optimize health and minimize adverse (side) effects^201^ (Fig. 3b). Melatonin supplementation can be used to restore the circadian rhythm of patients with circadian misalignment, thereby reducing the risk of CVDs^202,203^. In addition, controlled light systems in hospitals could also have beneficial effects. Hospitalization is known to weaken the circadian rhythmicity of patients^204^. A study by Mangini et al. established a protocol to successfully restore circadian rhythmicity in inpatients via light modulation using glasses that either allow or filter-specific wavelengths in the morning and evening, respectively^205^. Therefore, controlled light systems that can improve circadian rhythms should be considered in the clinic.

Different approaches to restoring circadian rhythmicity and reducing the risk of CVDs are based on other well-known *Zeitgebers*, such as food intake or exercise (Fig. 3b). The effects of food restriction were first tested in obese mice that were allowed to eat only during their active phase (nighttime) rather than during standard *ad libitum* feeding. This time-restricted feeding could restore the circadian pattern of cardiac triglyceride synthesis and partially reverse adverse cardiac remodeling in obese mice^140^. Time-restricted eating (TRE) has also been tested in humans, with a 4–10-h window for eating followed by 14–20 h of fasting. This regimen reduced fat mass, BP, and triglyceride levels and decreased cardiac oxidative stress in obese patients^206,207^. In addition, a recent study showed that firefighters subjected to TRE after 24 h of shift had improved cardiometabolic risk factors, such as decreased BP and HbA1c levels, suggesting that this strategy might be beneficial for treating or preventing cardiometabolic diseases^208^.

Physical exercise, another *Zeitgeber*, can be used to resynchronize the clock and prevent the risk of CVDs^209^. Moderate aerobic morning exercise helps reduce cardiovascular risk factors, such as high BP^210,211^. Thus, morning exercise could help to decrease the risk of non dipping hypertension induced by circadian misalignment, thereby reducing the risk of CVDs. However, this approach cannot be used in patients after heart surgery to avoid wound reopening or in patients with HF to prevent dangerous elevations in BP^212^. Nonetheless, physical exercise is a potent (re)synchronizer of the circadian clock and could help to prevent disease.

### Targeting the clock

Another arm of chronotherapy consists of targeting circadian clock proteins themselves with small molecules (Fig. 3c). The targets of these proteins include RORs, REV-ERBs, and CRY1/2, and both activating and inhibiting small molecules exist to modulate the function of these targets (e.g., SR9009, SR9011, SR8278, GSK4112, KL001, and nobiletin)^213–217^. Inhibiting REV-ERBs using SR8278 has emerged as a potential pharmacological approach for cardioprotection in cardiac injury mouse models^102,217^. In addition, the REV-ERB agonist SR9009 has been proven to have beneficial effects on cardiac remodeling after pressure overload^44,218–221^. Importantly, SR9009 was shown to have numerous transcriptional and metabolic effects independent of the circadian nuclear receptor REV-ERBα/β in noncardiac cell types^214,218^. Therefore, further characterization of clock-targeting molecules is needed to determine the mechanisms by which they exert their beneficial effects and whether these effects are a consequence of targeting clock proteins or off-target effects.

Targeting the clock could be beneficial for patients who are undergoing surgery in the morning to mimic the effects that patients who underwent surgery in the afternoon have and to reduce the risk of undesirable side effects occurring from morning surgery^102^. It could also reduce the risk of a morning infarct in high-risk patients, similar to what has been shown in *Clock*-mutant mice, which have a reduced risk of MI^92^. Another advantage of targeting the clock would be to temporarily ‘lock’ the clock in a certain phase to increase or decrease specific pathways that are beneficial for patients with CVD (Fig. 3c). For instance, FAO is often decreased in HF patients^222,223^, and CM-RevDKO mice exhibit a reduction in FAO^47^. Therefore, temporarily activating REV-ERBs could increase FAO levels and prevent the risk of CVDs. Importantly, the approach could be either short-term or combined with a procedure of well-tuned circadian rhythm maintenance to avoid adverse effects due to clock perturbation.

Altogether, although highly promising, pharmacological improvement and testing the specificity of these clock-targeting molecules are necessary before using them in the clinic for the treatment of a wide range of cardiovascular maladies.

## Conclusion and perspectives

The cardiac circadian clock plays an important role in both physiological and pathophysiological conditions. Most of our knowledge on the circadian clock stems from genetic mouse experiments, and human circadian studies are limited. Indeed, thus far, human studies have predominantly shown correlations between circadian rhythm perturbations and CVD induction. In this review, we highlight innovative approaches for studying the molecular mechanisms by which the circadian clock governs essential processes in the human heart. It will be imperative to focus on how desynchronization of the cardiac clock with its environment contributes to cardiovascular impairments. In addition, gaining knowledge on how the cardiac circadian clock can be leveraged to prevent and treat CVDs will be essential.
